# Effects of La Crosse virus infection on the host-seeking behavior and levels of two neurotransmitters in *Aedes triseriatus*

**DOI:** 10.1186/s13071-019-3658-6

**Published:** 2019-08-09

**Authors:** Fan Yang, Kevin Chan, Carlyle C. Brewster, Sally L. Paulson

**Affiliations:** 1 Department of Entomology, Virginia Tech, Blacksburg, VA USA; 2 Present Address: Vector-Borne Disease Section, California Department of Public Health, Richmond, CA USA

**Keywords:** La Crosse Virus, *Aedes triseriatus*, Host-seeking, Serotonin, Dopamine

## Abstract

**Background:**

La Crosse virus (LACV) infection has been shown to manipulate the blood-feeding behaviors of its main vector, *Aedes triseriatus*. Here, we investigated the effects of virus infection on serotonin and dopamine and their potential roles in host-seeking. In mosquitoes, serotonin depletion has been shown to interfere with blood-feeding but not host-seeking. Dopamine depletion does not affect either blood-feeding or host-seeking; elevations of dopamine, however, has been shown to inhibit host-seeking. The purpose of this study was to determine the effects of LACV infection on the host-seeking behavior of and neurotransmitter levels in *Ae. triseriatus*.

**Methods:**

Host-seeking behavior was evaluated using a uni-port olfactometer and a membrane feeder assay. Levels of serotonin and dopamine in infected and control mosquito heads were measured using HPLC-ED.

**Results:**

Infection with LACV significantly inhibited the activation and attraction of *Ae. triseriatus* females to a host. A higher proportion of uninfected *Ae. triseriatus* females were activated by the presence of a host compared to infected mosquitoes and more uninfected mosquitoes were full responders (95.7%) compared to infected ones (91.1%). However, infection with LACV did not significantly affect the landing, probing, or blood-feeding rates of female mosquitoes. LACV-infected mosquitoes had lower serotonin levels than controls (104.5 *vs* 138.3 pg/head) while the dopamine levels were not affected by infection status (282.3 *vs* 237 pg/head).

**Conclusions:**

Our work suggests that virus-induced reduction of serotonin is related to previously reported blood-feeding alterations in LACV-infected mosquitoes and could lead to enhanced transmission and increased vectorial capacity. In addition, some aspects of host-seeking were inhibited by virus infection.

## Background

La Crosse encephalitis (Family *Bunyaviridae*, California serogroup, LACV) is an important cause of arboviral neuroinvasive disease in the USA [1]. *Aedes triseriatus* (Say) is the primary vector to transmit this disease. The virus is zoonotic, maintained in nature through horizontal transmission to small woodland mammals, such as chipmunks and squirrels that act as amplifying hosts [2].

Pathogen-induced alterations of the blood-feeding behavior of insects resulting in enhanced transmission have been described for numerous parasite-vector systems [3]. For example, *Aedes aegypti* infected with dengue virus (DENV) displayed extended periods of probing compared to uninfected individuals [4]. Previous studies in our laboratory demonstrated that horizontal infection by LACV affected the blood feeding of *Ae. triseriatus* and *Aedes albopictus* mosquitoes [5]. Both species took smaller blood meals compared to uninfected siblings, and twice as many virus-infected *Ae. triseriatus* females fed multiple times in a 24-hour period compared with controls [5]. This virus-induced feeding alteration likely results in multiple host contacts within one gonotrophic cycle, thereby increasing transmission of LACV by its natural vector, *Ae. triseriatus*. However, little is known about the effects of virus infection on mosquito host-seeking behavior. Qualls et al. [6] found that *Ae. aegypti* with disseminated Sindbis virus infections took nearly 3 times longer to locate a blood meal and infection with DENV serotype-2 (DENV-2) significantly reduced the motivation of *Ae. aegypti* females to feed [7].

Many viruses have been shown to be neurotropic in the mosquito vector [8, 9] and several neurotransmitters have been found to play a role in controlling mosquito host seeking, biting and feeding behaviors. For example, elevation of dopamine levels can inhibit the host-seeking behavior of *Ae. albopictus* [10] while depletion of serotonin inhibited feeding by *Ae. triseriatus* [11]. Therefore, it is reasonable to hypothesize that virus-induced modulation of neurochemical levels may be a mechanism for altering blood feeding and/or host-seeking behaviors of infected mosquitoes. The purpose of this study was to determine if LACV infection affects host-seeking behavior of and neurotransmitter levels in *Ae. triseriatus*.

## Methods

### Virus isolates and assays

The VA0921075 isolate used in this study originated from adult *Ae. triseriatus* mosquitoes collected in 1999 in Wise County, VA, USA [12]. Prior to the study, the isolate was first amplified in adult female *Ae. triseriatus* and then on Vero cells. The titer of the stock virus was 2.05 × 10^8^ plaque forming units (PFU) /ml. Virus titers of the stock virus and individually infected mosquitoes were determined by plaque assay following the methods of Barker et al. [12].

### Mosquitoes

Eggs of *Ae. triseriatus* were collected from Blacksburg, VA, USA, in 2015. The eggs were hatched and held in an insectary maintained at 27.5 ± 1 °C, 75% relative humidity, and a 16:8 h L:D cycle. Because body size can influence host-seeking [13], mosquitoes used in this study were reared according to the methods of Jackson et al. [5] to ensure uniformity in adult size.

### Infection of mosquitoes

Three to five day-old unmated adult female mosquitoes were injected intrathoracically with 0.5 µl of LACV (2.05 × 10^8^ PFU) or M199 medium for infected and control groups, respectively following the methods of Jackson et al. [5]. After injection, mosquitoes were held under standard laboratory conditions for a 7-day extrinsic incubation period. During this time, they were provided with 10% sucrose *ad libitum*.

### Behavioral assays

Many different variables can influence host-seeking behavior, so care was taken to control for as many of these as possible. Because newly emerged females are not host responsive while older mosquitoes show increased host-seeking [14, 15], all trials were done on mosquitoes of the same age (10–13 days) to control for changes in host-seeking activity with age. Individuals vary in a heritable way in their attractiveness to mosquitoes [16, 17], so the same host was used in all trials. All trials were run at the same time of day to control for endogenous host-seeking rhythms [18].

### Long-range behavioral assay

**Fig. 1:**
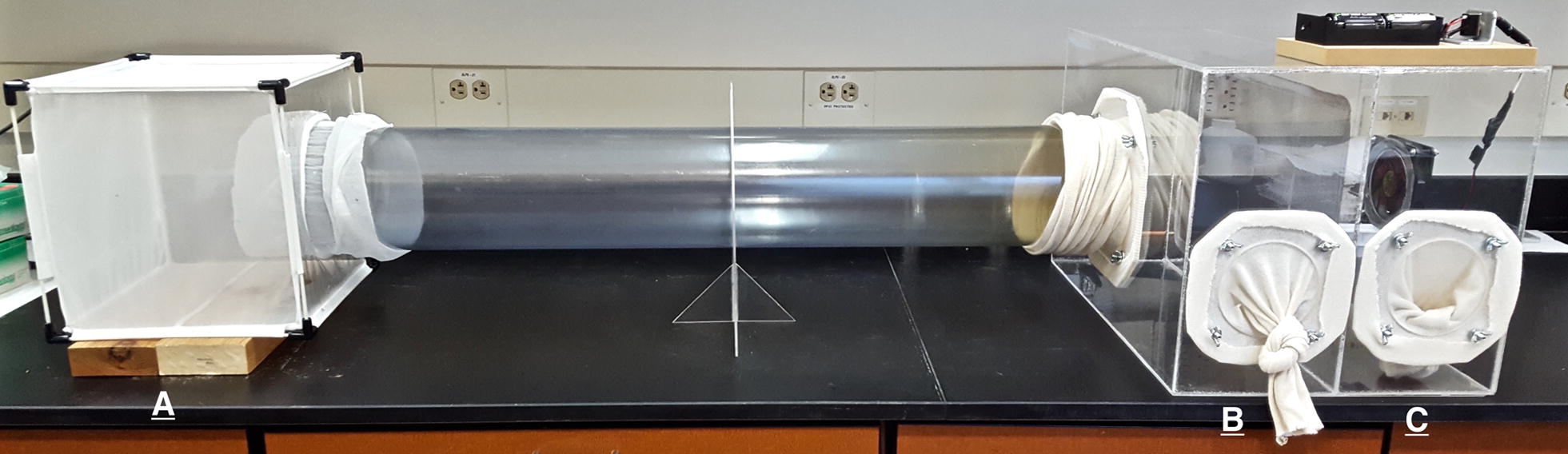
The uni-port olfactometer. The olfactometer consisted of a holding cage (**A**), a 1 m × 20 cm polystyrene tube, the mosquito trap (**B**), and the host compartment (**C**). Air flowed from right to left powered by a 12 V computer fan attached to the outside of the host compartment

Host-seeking was measured using a uni-port olfactometer modified from a design by Cabrini & Andrade [19]. The device consisted of a 30 cm^3^ holding cage, a 1 m × 20 cm polystyrene tube, and a 100 × 50 × 50 cm testing chamber (Fig. 1). The testing chamber was divided by a mesh partition into two parts: the collection chamber and the host compartment. A 12 V computer fan provided airflow from the attractant to the holding cage. Mosquitoes were aspirated into the holding cage and given a 30-min acclimatization period. Assays were run between 9:00 and 11:00 h because this is the peak time for feeding by *Ae. triseriatus* [20]. A human arm and breath introduced *via* a latex tube were the attractants [21]. The same host was used for each assay. Mosquito response was determined after a 10-min test period. Mosquitoes that left the holding chamber, travelled the length of the 1-m tube to enter the collection chamber at the end of the olfactometer tube were considered full responders, those that left the holding chamber but did not enter the collection chamber were scored as partial responders, and those that did not exit the holding chamber were non-responders. Any mosquito that left the holding chamber, whether it continued to the collection chamber or not, were said to be activated (full responders + partial responders). Activated mosquitoes that entered the collection chamber were considered attracted. The experiment was replicated 10 times for both infected and uninfected control mosquitoes. The mean number of mosquitoes in each trial was 90 for the infected and 85 for the control. To determine if virus titer varied in responding and non-responding mosquitoes, 10 mosquitoes from each category were randomly selected and assayed individually from 3 different trials.

### Short-range behavioral assays

To measure landing, probing and blood-feeding rates, groups of 30 infected or uninfected mosquitoes were placed in a plastic, cylindrical cage (11 cm high × 12 cm diameter) with a metal screen. A glass water-jacketed mosquito feeder containing defibrinated sheep blood (Colorado Serum Company, Denver, CO, USA) maintained at 37 °C was placed on the screen. Natural pork sausage casing was used as the membrane. Landing, probing, and blood-feeding events were recorded for 15 min during which time the observer was emanating human odor. Visible blood in the abdomen was considered positive for blood-feeding. Each experiment was repeated six times.

### Measurement of neurotransmitters

Two-week-old female mosquitoes from control and infected groups were frozen on dry ice for 10 min. Heads from control or infected groups were dissected at 9:00–11:00 h, the same time period as the host-seeking assays, and placed in groups of 5 for high performance liquid chromatography with electrochemical detection (HPLC-ED) measurement [22]. All samples were stored at − 70 °C immediately after collection. The heads were homogenized in 0.2 ml mobile phase pH 4.7 (sodium acetate 50 mM, citric acid 12.5 mM, EDTA 134 mM, octanesulfonic acid 230 mM, sodium chloride 2 mM, pH 4.7 and 12% methanol) by sonicator for 10 min on ice. Supernatant was collected by centrifuging homogenate for 15 min at 13,000× *rpm* at 4 °C and transferred into a new microcentrifuge tube for immediate analysis. The HPLC-ED system included the Agilent Technologies 1100 Series and an electrochemical detector (Waters 2465). Separation of electroactive species was achieved by a reverse-phase column (250 × 4.0, C18, with particle size 3 µM) with a flow rate of 0.5 ml/min. The working electrode was 0.8 V for serotonin and 0.6 V for dopamine *versus* an Ag/AgCl working electrode.

### Statistics

The number of mosquitoes activated and attracted were analyzed by two-sided Fisher’s exact tests to test for differences between virus-infected and uninfected mosquitoes. The whole body virus titers of responding and non-responding mosquitoes were compared by a two-tailed unpaired t-test. The number of mosquitoes landing, probing, and blood feeding in the membrane feeder assays were analyzed by two-sided Fisher’s exact tests. The levels of serotonin and dopamine in the heads of infected and uninfected mosquitoes were evaluated by two-tailed paired t-tests. For all analyses, an alpha of 0.05 was used as the cutoff for significance. All statistical analysis was done using Prism 7 for Mac OSX (GraphPad Software, Inc., 2017).

## Results

### Effect of virus status on activation and attraction

**Fig. 2:**
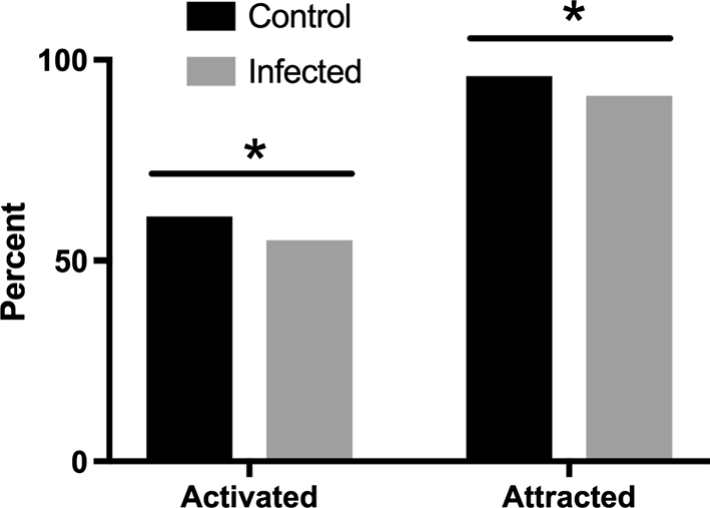
Effect of infection status on the rates of activation and attraction of mosquitoes in an olfactometer. Mosquitoes that exited the holding cage were considered to have been activated. Activated mosquitoes that travelled the length of the 1-m tube to enter the collection chamber at the end of the olfactometer were scored as attracted. *P < 0.05 (Fisher’s exact test)

**Fig. 3:**
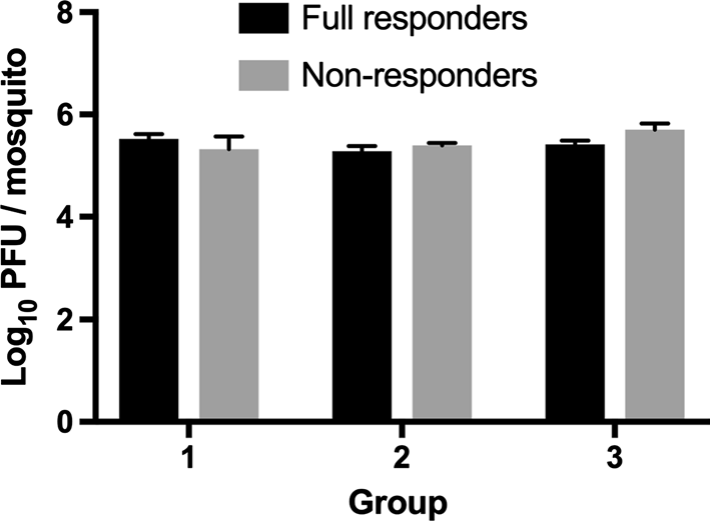
Mean (± SEM, *n* = 5) whole body LACV titers of full responders and non-responders. The level of virus titer in both responder and non-responder groups were equivalent (5.4 *vs* 5.5 log_10_ PFU/ mosquito) (*t* = 0.6042, *df* = 28, *P* > 0.05)

LACV infection had an inhibitory effect on the host-seeking behavior of female *Ae. triseriatus*. A higher proportion of uninfected *Ae. triseriatus* females were activated by the presence of a host compared to infected mosquitoes (60.9 *vs* 54.9%) (Fisher’s exact test, *P* < 0.05, OR: 1.27, 95% CI: 1.054–1.541) (Fig. 2). Although most activated mosquitoes were attracted and moved down the tube all the way to the collection chamber of the olfactometer regardless of infection status, a higher proportion of uninfected mosquitoes were full responders (95.7%) compared to 91.1% of infected mosquitoes (Fisher’s exact test, *P* < 0.01, OR: 2.19, 95% CI: 1.31–3.69) (Fig. 2). The level of virus titer in both responder and non-responder groups were equivalent (5.4 *vs* 5.5 log_10_ PFU/ mosquito) (*t* = 0.6042, *df* = 28, *P* > 0.05) (Fig. 3).

### Effect of infection status on landing, probing and blood-feeding

**Fig. 4:**
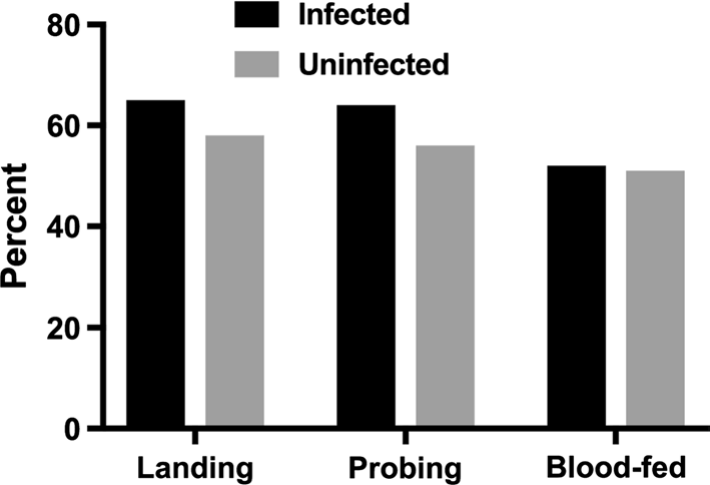
Proportion of infected and uninfected mosquitoes landing, probing and blood-feeding on a membrane feeder during a 15-minute test period. Infection with LACV did not significantly affect the landing, probing, or blood-feeding rates of female mosquitoes (Fisher’s exact test, *P* > 0.05)

Infection with LACV did not significantly affect the landing, probing, or blood-feeding rates of female mosquitoes (Fisher’s exact test, *P* > 0.05) (Fig. 4). During the 15-min test period, 65% of infected mosquitoes landed on the membrane surface *vs* 58% of the uninfected individuals (*P* = 0.12, OR: 1.57, 95% CI: 0.93–2.60), 64% of the infected mosquitoes probed the membrane compared to 56% of the uninfected ones (*P* = 0.07, OR: 1.64, 95% CI: 1.000–2.767), and 52% the infected group took a visible blood meal *vs* 51% of the uninfected individuals (*P* = 0.91, OR: 1.06, 95% CI: 0.67–1.66).

### Effect of virus infection on serotonin and dopamine levels in mosquito heads

**Fig. 5:**
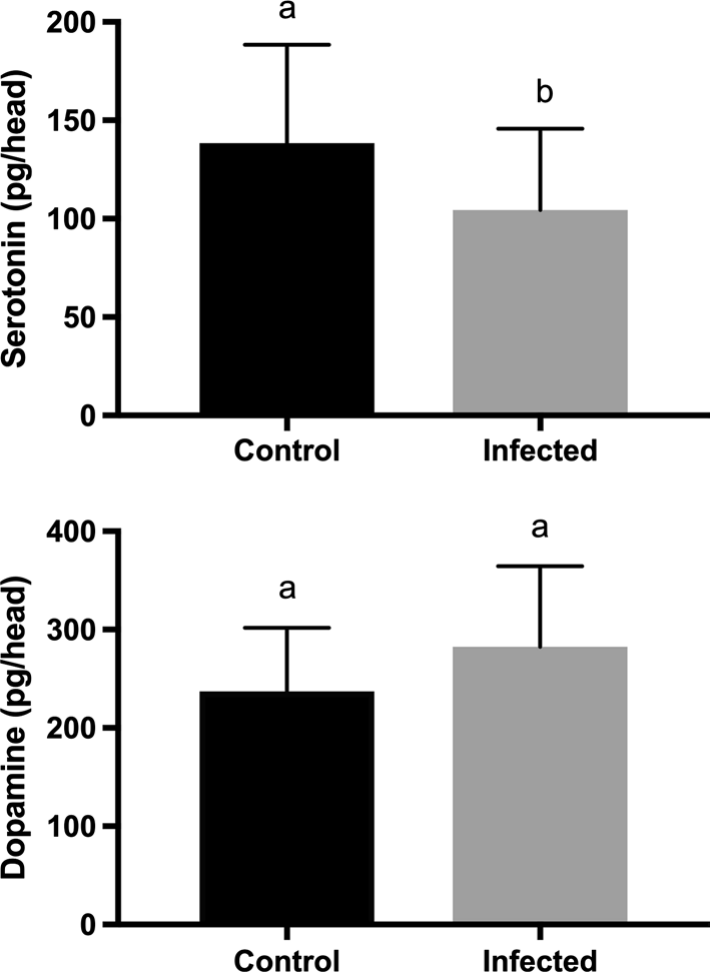
Mean (± SEM) serotonin and dopamine levels in infected and control mosquito heads. Heads from control or infected mosquitoes were dissected and placed in groups of 5 for high performance liquid chromatography with electrochemical detection (HPLC-ED) measurement. For each graph, columns with different letters are significantly different (paired t-test, *P* > 0.05)

The mean level of serotonin in the heads of infected female *Ae. triseriatus* was significantly lower compared with the heads of control individuals (104.5 *vs* 138.3 pg/head) (*t* = 5.685, *df* = 2, *P* < 0.05) (Fig. 5). However, levels of dopamine were not significantly different between infected and uninfected females (282.3 *vs* 237 pg/head) (*t* = 2.405, *df* = 2, *P* > 0.05) (Fig. 5).

## Discussion

This study showed that LACV infection inhibits the host-seeking behavior of *Ae. triseriatus* females. Hamilton & Hurd (2002) describe a 4-step model of blood-feeding behavior: (i) appetitive search; (ii) activation and orientation; (iii) attraction; and (iv) landing and probing [23]. Using an olfactometer, we were primarily measuring the second and third steps, i.e. activation and orientation and attraction, and found that these behaviors were inhibited by LACV infection. A combination of olfactory cues such as odor and CO_2_, and physical stimuli such as heat and color are important in the activation and attraction of mosquitoes to hosts [24–26]. There are hundreds of volatile compounds released in human breath and skin odor [27] but CO_2_ has been shown to act as a behavioral stimulator [28]. Visual cues are largely missing in the olfactometer assay, but the use of a human hand and breath would provide most of the other physical and chemical stimuli for activation and attraction. Using the membrane feeder assay, we found that the behaviors included in the fourth step, i.e. landing and probing, were not affected by LACV infection status. Heat and humidity, stimuli that are provided by the membrane feeder, are important cues determining whether or not a mosquito will land [29, 30]. Heat, odor and CO_2_ have been shown to act synergists to motivate mosquito probing and blood-feeding [28] all of which were provided during our assay.

A variety of pathogens have been shown to manipulate probing, engorgement and other feeding behaviors of mosquitoes to enhance transmission, behaviors that occur in close proximity to the host. However, few studies have examined the effect of infection on the earlier steps of blood-feeding that occur at a distance, such as the initiation of host-seeking and location of a host [23, 31]. For example, *Plasmodium gallinaceum-*infected *Ae. aegypti* were significantly more attracted to guinea pig odors compared to uninfected individuals [32] and *An. gambiae* infected with *P. falciparum* showed an increased attraction to human odors [33]. In a study of *An. stephensi* infected with *P. yoelii*, changes in attraction to a host were linked to changes in the responsiveness of the vector odorant receptors suggesting a possible neurophysiological mechanism [31]. Only a few studies have been done looking at the effect of virus infection on mosquito activation and/or attraction. For example, Qualls et al. [34] reported a significant increase in the activation times of *Ae. aegypti* infected with Sindbis virus but this work was done in a small cage (20 cm^3^) with a membrane feeder as an attractant rather than a living host. Female mosquitoes infected with West Nile virus showed a lower host-seeking response (attraction) using a 1.65 m one-port olfactometer [35] baited with 5% CO_2_ and socks with chicken or human odor. We tested host activation and attraction over a distance of 1 m using a host frequently fed upon by *Ae. triseriatus* in nature (human hand and breath). So despite different viruses used, in all three studies mentioned above, the early steps of host-seeking, i.e. activation and attraction, were inhibited by virus infection.

The stimuli that control landing act over a short range and include factors such as odor, heat, visual and moisture cues. Probing and feeding, on the other hand, are dependent on the interaction between the host and vector [23]. We did not observe any effect of virus infection on landing, probing, or blood-feeding in this study. However, we did not measure the amount of blood imbibed but instead tested whether or not blood was obtained, regardless of amount. This is in contrast to a previous study that showed that LACV-infected mosquitoes took smaller blood meals and were more likely to feed multiple times compared to uninfected mosquitoes [5]. A study by Maciel-de-Freitas et al. [7] also showed that dengue virus-infected *Ae. aegypti* were more likely to re-feed than uninfected individuals. Thus, virus infection can affect mosquito blood-feeding in a way that could increase vectorial capacity.

The mechanism by which a pathogen enhances its transmission by a mosquito is unclear. Insect behavior is mainly driven by rewards and punishments, which are organized by a network of interacting circuits of several biogenic aminergic neurons [36]. Biogenic amines can act as neurotransmitters, neuromodulators or neurohormones in insects. The amines serotonin (5-HT) and dopamine act to control and regulate physiological functions such as circadian rhythms, endocrine secretion, cardiovascular control and even learning and memory [37]. Several studies have indicated a role of serotonin in controlling blood-feeding by mosquitoes. The salivary glands of female *Ae. aegypti* demonstrate 5-HT-immunoreactive innervation, which is absent in male salivary glands [38]. Also, when treated with *a*-methyl-tryptophan (AMTP, a chemical that depletes serotonin when injected into a mosquito), females secreted less saliva and that saliva contained less apyrase than control mosquitoes [38]. Apyrase is an enzyme that inhibits ADP-dependent platelet aggregation, thus facilitating blood intake [39]. The AMTP treated mosquitoes probed longer and showed a lower blood-feeding success. In a similar study using *Ae. triseriatus*, AMTP treatment resulted in significantly reduced blood-feeding success but the host-seeking ability was not altered [10]. Dopamine does not seem to be involved in controlling blood-feeding but rather host-seeking. Injecting *a*-methyl-tyrosine (AMT), which causes dopamine reduction but does not affect serotonin, into *Ae. triseriatus* did not affect blood-feeding or host-seeking [10]. However, elevation of dopamine levels reduced host-seeking activity in *Ae. albopictus* [9].

## Conclusions

In this study, we found that LACV-infected mosquitoes had lower serotonin levels than controls, while dopamine levels were not affected. This virus-induced reduction of serotonin may be related to the blood-feeding alteration exhibited by LACV-infected mosquitoes reported by Jackson et al. [5]. Infected mosquitoes took smaller blood meals and fed more frequently than uninfected females resulting in enhanced transmission and increased vectorial capacity. Importantly, we showed that landing and probing rates and ability to locate blood are not affected by LACV infection, permitting virus transmission to occur. However, inhibition of host-seeking activity could offset the enhancement of virus transmission through changes in blood-feeding ability. It is interesting to note that several viruses in the family Bunyaviridae have been shown to affect the feeding behavior of the vector including LACV [5], Rift Valley fever [40] and tobacco spotted wilt virus [41]. Han et al. (2015) speculated that this might be a conserved trait among the bunyaviruses [42]. Thus, it is possible that bunyaviruses exert an effect on the levels of biogenic amines in the vector, promoting virus transmission through altered blood-feeding with only slight impairment of the vector’s ability to locate a host.

## Data Availability

The datasets used for the present study are available from the corresponding author upon request.

## Abbreviations

LACV: La Crosse virus
DENV: Dengue virus
PFU: plaque forming units
HPLC-ED: high performance liquid chromatography with electrochemical detection
